# Increased matrix metalloproteinase activation in esophageal squamous cell carcinoma

**DOI:** 10.1186/1479-5876-8-91

**Published:** 2010-10-05

**Authors:** Sumana Mukherjee, Mark J Roth, Sanford M Dawsey, Wusheng Yan, Jaime Rodriguez-Canales, Heidi S Erickson, Nan Hu, Alisa M Goldstein, Philip R Taylor, Annely M Richardson, Michael A Tangrea, Rodrigo F Chuaqui, Michael R Emmert-Buck

**Affiliations:** 1 Pathogenetics Unit, Laboratory of Pathology, National Cancer Institute, National Institutes of Health, Bethesda, MD USA; 2 Nutritional Epidemiology Branch, Division of Cancer Epidemiology and Genetics, National Cancer Institute, National Institutes of Health, Bethesda, MD USA; 3 Genetic Epidemiology Branch, Division of Cancer Epidemiology and Genetics, National Cancer Institute, National Institutes of Health, Bethesda, MD USA

## Abstract

**Background:**

Esophageal squamous cell carcinomas (ESCC) are usually asymptomatic and go undetected until they are incurable. Cytological screening is one strategy to detect ESCC at an early stage and has shown promise in previous studies, although improvement in sensitivity and specificity are needed. Proteases modulate cancer progression by facilitating tumor invasion and metastasis. In the current study, matrix metalloproteinases (MMPs) were studied in a search for new early detection markers for ESCC.

**Methods:**

Protein expression levels of MMPs were measured using zymography in 24 cases of paired normal esophagus and ESCC, and in the tumor-associated stroma and tumor epithelium in one sample after laser capture microdissection (LCM). MMP-3 and MMP-10 transcripts in both the epithelium and stroma in five cases were further analyzed by quantitative reverse transcriptase polymerase chain reaction (qRT-PCR).

**Results:**

Gelatin zymography showed bands corresponding in size to MMP-2, MMP-3, MMP-9, and MMP-10 enzymes in each of the 24 cancer cases. MMP levels tended to be higher in tumors than paired normal tissue; however, only the 45 kDa band that corresponds to the activated form of MMP-3 and MMP-10 was strongly expressed in all 24 tumors with little or no expression in the paired normal foci. LCM-based analysis showed the 45 kDA band to be present in both the stromal and epithelial components of the tumor microenvironment, and that MMP-3 and MMP-10 mRNA levels were higher in tumors than paired normal tissues for each compartment.

**Conclusions:**

Increased levels of MMPs occur in ESCC suggesting their up-regulation is important in esophageal tumorigenesis. The up-regulated gene products have the potential to serve as early detection markers in the clinic.

## Background

Esophageal cancer is the sixth leading cause of cancer death in the world [1]. Eighty percent of esophageal cancer cases occur in developing countries, and in these areas about 90% are esophageal squamous cell carcinomas (ESCC) [2]. In high-risk areas, such as Linxian, China, ESCC is the leading cause of cancer death with mortality rates in excess of 100/100,000 people per year in both sexes [3]. Clinically, ESCC is characterized by rapid progression and poor prognosis. Patients with Stage I tumors (T1N0M0), invading only the lamina propria or submucosa without lymph node or distant metastasis [4], have a 90% 5-year survival after resection, but only 1% of patients are diagnosed with Stage I disease [5]. A significant reduction of ESCC mortality will require development of new drugs for advanced tumors and/or new strategies for early detection and treatment of precursor lesions and early cancers.

Endoscopy with iodine staining is an accurate way to identify and localize precursor and early invasive lesions of ESCC [6], but this procedure is too invasive and expensive to serve as a primary screening exam, even in very high-risk populations. After proper diagnosis, surgical treatments are available that are safe and effective, thus there is a need for screening approaches suitable for population- and clinic-based assays for early detection that can identify patients for follow-up endoscopic examination. Esophageal balloon cytology (EBC) examination is one such approach for ESCC screening; however, previous studies have shown that morphologic diagnosis of the collected cells is not sufficient due to a sensitivity/specificity of only 46%/84% for biopsy-proven squamous dysplasia or cancer and therefore a supplemental molecular test for EBC is needed [7]. MMPs are elevated in many cancers and immunohistochemistry-based studies have been reported showing MMP increases in ESCC, thus they are attractive candidates for evaluation as potential ancillary molecular markers [8-13]. To date, though, a comprehensive profile of MMP levels and activation status in ESCC has not been performed. The aim of this study was to assess MMPs in ESCC as potential clinical markers of tumorigenesis, using a highly sensitive zymography method capable of measuring both the inactive pro-forms and active forms of the enzymes.

## Methods

### Tissue Samples

All cases and samples were obtained from subjects residing in the Taihang mountain region of north central China. The study was approved by the Institutional Review Boards of the collaborating institutions: Shanxi Cancer Hospital and Institute, Taiyuan, Shanxi Province, China; and the National Cancer Institute, Bethesda, MD, USA.

Resection specimens from 24 ESCC patients (for clinical data refer to Table 1) treated at the Shanxi Cancer Hospital in Taiyuan, Shanxi Province were blocked and stored at -70°C until assays could be performed. Serial 8-micron frozen sections were cut from each tissue block using a Leica Cryostat and representative foci of patient-matched normal mucosa (N = 24) and invasive squamous cell carcinoma (N = 24) were chosen based on histological review of hematoxylin-and-eosin-stained slides by two pathologists (J.R.C. and R.F.C.) using accepted criteria.

**Table 1:** Clinical data

No.	Age	Sex	Smoking	Alcohol	Diagnosis	Tumor stage	Tumor grade	LN metastasis
1	55	Female	No	No	SCC	1	2	Yes
2	56	Male	Yes	Yes	SCC	3	1	Yes
3	55	Male	Yes	No	SCC	2	2	Yes
4	61	Male	No	No	SCC	3	2	Yes
5	52	Male	Yes	No	SCC	2	1	Yes
6	48	Female	Yes	No	SCC	3	2	No
7	missing				SCC	missing		
8	missing				SCC	missing		
9	67	Female	Yes	No	SCC	3	3	Yes
10	missing				SCC	missing		
11	65	Female	No	No	SCC	2	1	No
12	51	Male	Yes	No	SCC	3	2	No
13	56	Female	No	No	SCC	3	2	Yes
14	50	Male	Yes	No	SCC	2	3	Yes
15	missing				SCC	missing		
16	40	Male	Yes	No	SCC	3	2	Yes
17	62	Female	Yes	No	SCC	3	2	No
18	63	Male	No	No	SCC	3	1	No
19	70	Male	Yes	No	SCC	3	2	No
20	62	Male	Yes	No	SCC	3	2	No
21	missing				SCC	missing		
22	68	Male	Yes	No	SCC	3	2	Yes
23	63	Male	Yes	No	SCC	3	2	No
24	61	Male	Yes	No	SCC	3	2	No

### Gelatin Zymography

Gelatin zymography was performed as previously described with some modifications [14]. 10 μl of tissue lysate containing 8 μg of protein, determined using the Micro BCA™ Protein Assay kit (Thermo Scientific/Pierce, Rockford, IL), was mixed with an equal volume of Novex^® ^Tris-glycine SDS native sample buffer (Invitrogen™ Carlsbad, CA, USA) and the mixture was loaded into wells of pre-cast 10% Novex^® ^zymogram gelatin gels (Invitrogen™). Pre-stained molecular weight standards were also run on each gel. The gels were electrophoresed at a constant voltage of 125 V for approximately 2 h.

Following electrophoresis, the gels were rinsed in distilled water and then gently shaken in a renaturing solution of 2.7% Triton X-100 (Novex^® ^zymogram renaturing buffer, Invitrogen™) for 1 h at 37°C to reactivate MMPs. The gels were then incubated on a rotary shaker in a developing buffer (Novex^® ^zymogram developing buffer, Invitrogen™) for 24 h at 37°C to allow denatured MMPs to digest the gelatin substrate. After the digestion phase, the gels were rinsed and stained by incubation with Coomassie Blue Rapid stain (Diversified Biotech, Boston, MA, USA) for 1 h. Gels were destained with a solution of acetic acid, methanol and water (10: 50: 40) to maximize contrast between proteolytic areas and non-digested areas. Proteolytic activity was visualized as areas of clear bands against a dark blue background. The identity of the proteases was determined by analysis of the distance that the bands migrated on the gels, compared with the distance for migration of molecular weight standards.

### Laser Capture Microdissection

Serial frozen 8-μm sections were cut using a Leica Cryostat and placed onto uncharged glass slides. Every sixth slide was stained using hematoxylin-and-eosin and the histology confirmed by a pathologist (R.F.C. or J.R.C.). The remaining slides were stored at -80°C, not to exceed two weeks prior to dissection. The slides were placed on dry ice and then were stained as follows: 70% ethanol for 15 seconds, Mayer's hematoxylin (Sigma-Aldrich, St. Louis, MO) for 15 seconds, deionized water and bluing solution (Sigma-Aldrich) for 10 seconds each, and eosin (Sigma-Aldrich) for five seconds followed by dehydration using increased concentrations of ethanol (95%, 95%, 100% and 100%) for 10 seconds each. Tissue was then placed in xylenes for 20 seconds to complete the dehydration process.

LCM was performed using the PixCell IIe (Arcturus Engineering, Inc., Mountain View, CA) to isolate neoplastic epithelium and tumor stroma separately. Tumor-associated stromal fibroblasts and matrix were collected from locations proximate to epithelial tumor cells, being within 5 mm of an epithelial tumor nodule. Normal epithelial and stromal cells were similarly collected from histologically normal tissues. The time from slide removal from dry ice to completion of LCM did not exceed 30 minutes. On average, epithelial dissections required 3,000 shots (laser spot specifications: 30 μm spot size, 45-55 mW power, 3.0-4.0 ms duration); whereas stromal dissections required 4000 - 5000 shots.

### Quantitative RT-PCR

Total RNA was isolated with the PicoPure RNA Isolation kit (Arcturus Engineering) as suggested by the manufacturer. RNA quantity was assessed using NanoDrop Spectrophotometer (NanoDrop Technologies, Wilmington, DE). RNA quality, both 28S/18S ratio and RNA integrity number (RIN), was measured using the 2100 Bioanalyzer (Agilent Technologies, Inc., Palo Alto, CA) (Table-2).

Total RNA was used to generate complementary DNA (cDNA) using the Taqman High Capacity cDNA Reverse Transcription kit (Applied Biosystems, Inc., Foster City, CA, USA Cat # 4374966) as suggested by the manufacturer to get the maximum expression of transcripts. Singleplex qPCR was performed after first strand cDNA synthesis using 2× Taqman Universal PCR Master Mix (Applied Biosystems, Inc., Cat#4364338) and Amplitaq Gold DNA polymerase, LD (Applied Biosystems, Inc., Cat#4338857) and specific primer/probe sets (Applied Biosystems, Inc.). Five cases were tested with commercially available optimized primer/probe sets for MMP-3 [TaqMan Gene Expression Assays, Inventoried Assay ID: Hs00233962 for MMP-3 (stromelysin-1, progelatinase), Applied BioSystems, Inc., Cat.# 4331182] and MMP-10 [TaqMan Gene Expression Assays, Inventoried Assay ID: Hs00233987 for MMP-10 (stromelysin-2), Applied BioSystems, Inc., Cat.# 4331182] gene expression levels. All primer and probe sets are cDNA specific. All qPCR assays were performed in triplicate after reverse transcription. Beta-actin (ACTB), a known housekeeping gene, was used for normalization. Taqman primer/probe sets and master mix reagents were procured from Applied Biosystems (Foster City, CA).

Each reaction was conducted in a 20 μl volume using Applied Biosystems 7500 Real-Time PCR system (Foster City, CA). Cycling conditions consisted of one cycle of 50°C for 2 min followed by 95°C for 10 min, and then 50 cycles of 95°C for 15 seconds followed by 60°C for 1 min. Controls consisting of total human esophageal RNA (100 ηg/μl; Ambion, Austin, TX, USA) were positive in all runs, and controls consisting of sterile molecular grade water were negative in all runs. Critical threshold (Ct) cycle numbers were obtained for amplification of MMP-3, MMP-10, and ACTB. ΔCt values were calculated by subtracting the average Ct value of ACTB from the average Ct value of MMP-3 and MMP-10 in each case. Relative quantitation analysis of gene expression data was conducted according to the 2^-ΔΔCT ^method [15].

## Results and Discussion

Increased expression of matrix metalloproteinases (MMPs) are observed in many normal physiological processes and in several tumor types [16-25]. MMPs serve numerous and diverse functions, are under tight cell type-dependent control, and are normally expressed at low levels. However, when tissue remodeling occurs, such as in inflammation, wound healing, or cancer, MMPs are rapidly transcribed, secreted, and activated. In cancer, the enzymes have been shown to play a role in multiple steps of tumor progression including angiogenesis, local invasion, tumor cell intravasation and extravasation, and formation of distant metastases. The transcription of MMPs is induced by a variety of growth factors and most MMPs are secreted as inactive pro-enzymes that are activated either by cleavage through other proteinases or by induction of autocatalytic processing. Several studies suggest that there may be organ or cell type specificity associated with the up-regulation of proteolytic activity during malignant conversion.

**Figure 1:**
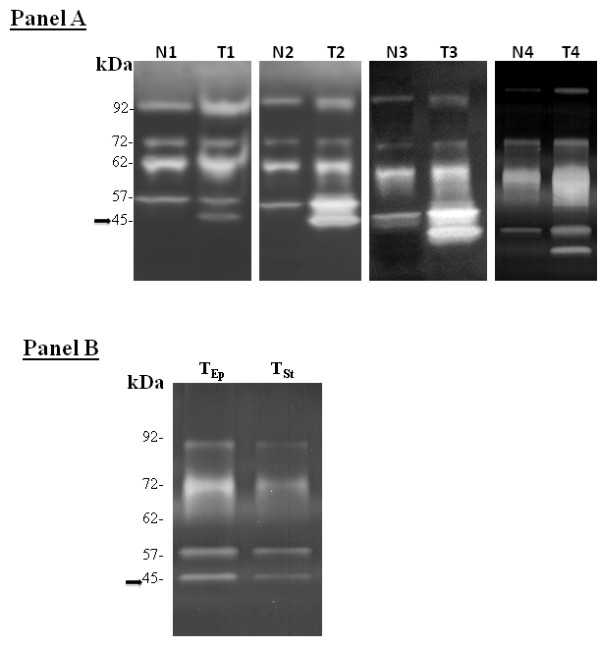
**Determination of MMP Levels.** (A) Zymogram measurement of MMPs in matched normal esophagus (N) and ESCC (T) in four cases. (B) Zymogram measurement of MMPs in the epithelial (T_Ep_) and stromal (T_St_) compartments in ESCC after procurement by LCM.

In the present study, MMP levels were assessed in ESCC in a search for molecular markers that could serve as useful adjunct tests for EBC screening. The primary finding was that a 45 kDA band corresponding in size to the activated form of MMP-3 and/or MMP-10 (stromelysin 1 and stromelysin 2, respectively) protein showed significant tumor-related up-regulation in all 24 patients specimens studied. As seen in Panel A of the Figure 1, four representative cases show a strong 45 kDa in each of the tumors with little or no expression in the normal samples. The 45 kDA band was not observed in 21 of the 24 normal esophageal specimens and a faint band was seen in three of the normals. A 57 kDa band corresponding in size to the pro-enzyme form of MMP-3/MMP-10 also showed tumor up-regulation; however, the band was also present at relatively high levels in the normal samples. Twenty-two of the 24 cases showed over-expression of the 57 kDa pro-enzyme in tumors with an overall increase of approximately two-fold.

In contrast, bands corresponding in size to MMP-2 and MMP-9 showed less consistent increases in ESCC. Zymographic analysis revealed that pro-MMP-2 (72 kDa) was up-regulated in 16 the 24 tumors compared to normal. Activated MMP-2 (62 kDa) was observed in all of the normal epithelium and tumor foci, with increased levels in 11 out of 24 tumors (45%). Activated MMP-9 (82 kDa) was not seen in any of the esophageal samples, but the expression of pro-MMP-9 (92 kDa) was elevated in 18 out of 24 tumors (75%) in the study.

To assess MMP expression and activation state selectively in the epithelial and stromal compartments within the tumor microenvironment, the enzymes were specifically measured in one case following microdissection. Zymographic analysis demonstrated that pro and active MMP-3/MMP-10 were present in both the stroma and epithelium (Figure 1B), indicating that further study of the genes, via such techniques as qRT-PCR measurement, should include both dissected epithelium and stroma in the normal and cancerous specimens.

We could not distinguish the specific identity of the up-regulated 45 kDA band since the activated forms of MMP-3 and MMP-10 migrate together during gel electrophoresis, and immunoblot analysis was unsuccessful due in part to the limited amount of available clinical material. Thus, MMP-3 and MMP-10 were assessed at the transcript level using qRT-PCR. Both the epithelial and stromal compartments were analyzed. LCM was performed for 10 tissue blocks (5 cases of matched normal and tumor) and approximately 10,000 dissected cells were procured from the epithelium and the stroma from each block (Table 2). Total RNA was used to generate cDNA and then quantitative real-time PCR (qPCR) gene expression measurements were performed and normalized to that of ACTB mRNA [15]. The average Ct values for the normal epithelium and stromal compartments were in the range of 35-40 and in some cases were undetected after 50 cycles; whereas, the tumor epithelial and stromal compartments showed a Ct value in the range 20-30 for both MMP-3 and MMP-10 mRNA, significantly more than in the counterpart normal cells (Table 3). These data support the notion that the tumor up-regulated 45 kDa band observed by zymography is due to both MMP-3 and MMP-10 enzymes.

**Table 2:** RNA preparation and assessment

Case	Histology	LCM shots	Nano-drop (ηg/μl)	RIN
1	Normal Epithelium	4000	14.2	6.1
	Normal Stroma	5000	11.5	2.7
	Tumor Epithelium	4500	23.1	4.8
	Tumor Stroma	10000	45.5	3.7
2	Normal Epithelium	3000	2.0	5.0
	Normal Stroma	6000	2.2	7.3
	Tumor Epithelium	3000	2	6.5
	Tumor Stroma	6000	1	4.1
3	Normal Epithelium	3000	3.63	4.1
	Normal Stroma	5000	5.06	7.0
	Tumor Epithelium	3000	3.02	7.0
	Tumor Stroma	5000	5.67	6.2
4	Normal Epithelium	3000	5.08	4.7
	Normal Stroma	2000	1.28	7.4
	Tumor Epithelium	3000	4.37	8.0
	Tumor Stroma	4000	2.05	5.9
5	Normal Epithelium	2500	2.34	7.0
	Normal Stroma	4000	2.19	6.5
	Tumor Epithelium	2500	2.57	5.8
	Tumor Stroma	3000	1.76	5.8

**Table 3:** Gene expression comparison of normal epithelium versus tumor cells, and normal stroma versus tumor stroma

Case	Sample	Detector	Avg.Ct	St. Dev	ΔCtN	Sample	Av.Ct	St. dev	ΔCtT	ΔΔCt	2^^^-ΔΔCt
1	NE	MMP3	39.59		17.86	TE	25.50	0.108	5.37	-12.48	↑
	NE	MMP10	38.59	3.43	16.85	TE	21.39	0.07	1.25	-15.59	↑
	NE	ACTB	21.73	0.22		TE	20.13	0.03			
	NS	MMP3	36.20	0.75	15.77	TS	28.79	0.19	9.3	-6.47	↑
	NS	MMP10	35.38	0.75	14.95	TS	22.35	0.07	2.85	-12.09	↑
	NS	ACTB	20.43	0.06		TS	19.49	0.04			
2	NE	MMP3	39.54		13.77	TE	39.87		12.29	-1.48	↑
	NE	MMP10	38.68		12.92	TE	30.25	0.02	2.66	-10.25	↑
	NE	ACTB	25.76	0.166		TE	27.58	0.22			
	NS	MMP3	39.97		15.03	TS	32.58	0.42	4.21	-10.82	↑
	NS	MMP10	UD			TS	31.02	0.17	2.65	OFF-N, ON-T	↑
	NS	ACTB	24.93	0.051		TS	28.36	0.159			
3	NE	MMP3	UD			TE	38.51	1.49	14.13	OFF-N, ON-T	↑
	NE	MMP10	UD			TE	31.93	0.257	7.55	OFF-N, ON-T	↑
	NE	ACTB	23.45	0.058		TE	24.38	0.07			
	NS	MMP3	UD			TS	36.23	1.1	11.44	OFF-N, ON-T	↑
	NS	MMP10	UD			TS	34.13	0.80	9.34	OFF-N, ON-T	↑
	NS	ACTB	24.40	0.016		TS	24.79	0.07			
4	NE	MMP3	UD			TE	31.89	0.174		OFF-N, ON-T	↑
	NE	MMP10	39.42		15.99	TE	27.00	0.03	4.59	-11.40	↑
	NE	ACTB	23.43	0.056		TE	22.41	0.033			
	NS	MMP3	38.95		15.91	TS	29.61	0.108	5.24	-10.66	↑
	NS	MMP10	39.1		16.06	TS	27.69	0.118	3.32	-12.73	↑
	NS	ACTB	23.03	0.095		TS	24.36	0.05			
5	NE	MMP3	UD			TE	29.94	0.172		OFF-N, ON-T	↑
	NE	MMP10	UD			TE	30.28	0.209		OFF-N, ON-T	↑
	NE	ACTB	25.31	0.073		TE	25.08	0.048			
	NS	MMP3	39.65		16.22	TS	32.59	0.334	4.81	-11.40	↑
	NS	MMP10	39.38		15.95	TS	33.01	0.451	5.23	-10.72	↑
	NS	ACTB	23.43	0.0013		TS	27.78	0.0911			

NE, normal epithelium; NS, normal stroma; TE, tumor epithelium; TS, tumor stroma.

## Conclusions

In summary, the present study showed an increase in a band corresponding in size to active MMP-3/MMP-10 protein, and elevated MMP-3 and MMP-10 mRNA in the ESCC microenvironment, suggesting the enzymes play an important role in the disease process. The advantages of zymographic analysis include low-cost and simplicity, and the analysis requires little or no instrumentation since the activated MMPs migrate as unique bands. Equally important is that zymograms utilize the catalytic nature of MMPs for detection, thus the assay is extremely sensitive. The combination of a tumor-unique signal and an enzyme-based assay producing high sensitivity makes zymography a good candidate technology as an adjunct molecular screening tool for ESCC. Up-regulation of MMP-3/10 warrants further investigation as a potential diagnostic marker in the clinic.

## Competing interests

The authors declare that they have no competing interests.

## Authors' contributions

SM participated in the design of the study, worked up the ESCC cases; supported data analysis and drafted the manuscript. MJR was involved in study design and drafted the manuscript. SMD was involved in study design, drafted the manuscript, and provided ESCC cases. WY was involved in RNA analysis. JRC was the pathologist and evaluated the histopathology of the cases. HSE was involved in the RNA analysis and additional technical assistance. NH, AMG, PRT coordinated the study and drafted the manuscript. AMR supported the data with zymogram analysis. MAT helped in drafting the manuscript. RFC participated in the study design and added technical support. MRE-B participated in the design of the study, helped analyze the data, and was involved in writing and editing the manuscript. All authors read and approved the final manuscript.
